# Edwin Welse Coathupe

**Published:** 1926

**Authors:** 


### EDWIN WELSE COATHUPE, M.R.C.S.

The death of Mr. Edwin Welse Coathupe at the age of 89 at Boscombe, Bournemouth, on November 23rd, 1926, recalls to memory one of the most interesting careers in the history of the Bristol Medical School. Born in 1837 at Birdcombe Court, Wraxall, he was a son of Mr. Charles Thornton Coathupe, who was one of the Charity Trustees for Bristol, and also a representative in the City Council. His early tuition was received at the Grand Ducal College of Darmstadt, and subsequently he was educated at the well-known school in Clifton conducted by the Rev. J. Exley, M.A. He afterwards entered as a pupil at the Medical School and Royal Infirmary at Bristol, obtained the diploma of membership of the Royal College of Surgeons in 1859, and almost immediately accepted the appointment of Assistant Surgeon to the Tredegar Iron Works, which he held for three years.

As a student in Bristol and in London, whenever he had the chance he went about with the police, and especially when there was a difficult case he was admitted among the detectives, and actively joined in their work. He was always held by them in high esteem, and at an early age he gave up his work in Tredegar to join the Metropolitan Police as a constable. He was at once appointed into the inner circle of the Metropolitan detective force, consisting of some dozen members only.

After three years' police service in London he returned to Tredegar as chief surgeon at a salary of £1,000 a year. A year later he retired from the practice of medicine in order to become deputy Chief Constable of Manchester, and in 1876 Bristol was very fortunate in selecting him from 122 candidates as Chief Constable, and in retaining his very valuable services for many years.

Mr. Richardson Cross, who knew Mr. Coathupe very well, writes: "Soon after I came to Bristol (1878) the death of Mr. Tibbits, one of the Surgeons to the Infirmary, made a vacancy for the post of Surgeon to the Police, and to this I was appointed. The Chief Constable, Mr. Coathupe, then became intimately known to me. I held the highest possible regard for him in every way, both in his private life and for the high standard of his public work. Everyone looked up to him, the Watch Committee, the public, and the police themselves. He expected everyone in the Force to work up to his own standard—the best he could do. His desire was to improve each man, and also the whole body of the Force. I remember in physique we had got such a fine body of men, that a special effort to send a body of high-class constables to China resulted in a draft from Bristol of, I think, fifty of our men *en bloc*. He was interested in the medical men and in their work, and he often attended our meetings when anything came on of especial interest. But the detection of crime and the capture of the criminal was his absorbing interest, and he told me many stories of his adventures with the police in London and of his thrilling personal experiences."

Under his rule our police force doubled in number and its constitution was entirely remodelled. It was upon his initiative that the city was divided into divisions, the rank of Superintendent created, the Police Fire Brigade established, and the River Police brought into existence. He also introduced the general use of police vans. Under Mr. Coathupe's control the reputation of the force was steadily advanced, a fact which was shown by the number of men trained under him who secured high positions in various parts of the country.

Upon his retirement in 1894 he was presented with an address and £260 by the Justices of Bristol.

Thus, after 30 years' police service he sought well-earned rest. For a time he lived abroad, principally in Rome, but latterly he made his home at Bournemouth, where he died.

He had one child, a daughter, whose death in her twenties was a great sorrow to him.

His funeral took place at Wraxall, near his father's old home. His widow survives him and was present at his funeral. He had outlived most of his friends, but some of his old friends' children attended his funeral, together with several of his old superintendents in the police force, who had come long distances to pay their tribute to his memory.

We offer to Mrs. Coathupe our deep sympathy in her loss, together with the assurance that his name will not be forgotten in the list of those who have brought honour to our profession and our School.

